# Residual stress determination by the layer removal and X-ray diffraction measurement – correction method

**DOI:** 10.1016/j.mex.2022.101768

**Published:** 2022-06-22

**Authors:** Pavol Dlhý, Jan Poduška, Pavel Pokorný, Michal Jambor, Luboš Náhlík, Pavel Hutař

**Affiliations:** Institute of Physics of Materials, Czech Academy of Sciences, Žižkova 22, 616 00 Brno, Czech Republic

**Keywords:** Residual stress determination, X-ray diffraction, Cylindrical component, Analytical solution, Railway axles

## Abstract

Very often a manufacturing process is followed by some surface treatment. Such a process induces residual stress into the manufactured component. Compressive residual stress is desirable for enhancing the fatigue properties of the component. The residual stress is often measured only at the surface, if at all. However, residual stress is equilibrating in the whole component. Therefore, compressive residual stress at the surface induces undesirable tensile stress inside in the component. Knowledge of the residual stress distribution in a body can be very useful in engineering applications. The authors found this knowledge necessary for a proper description of fatigue crack propagation in railway axles described in the original paper [1]. With the onset of modern surface treating technologies, e.g. induction hardening, which can affect the entire cross-section of the component, the residual stress determination is even more critical.

The presented paper aims to describe a procedure developed for proper residual stress determination. The procedure can be easily used e.g. in R&D centers, where X-ray diffraction residual stress measurement is already in use. The procedure is suitable for the residual stress determination in sizable cylindrical bodies or components, e.g. railway axles. It uses X-ray diffraction residual stress surface measurement and layer removal by machining. Results experimentally obtained are corrected by a general procedure developed in MATLAB software in order to obtain the original residual stress state in the cylindrical body.

• More accurate procedure for a residual stress determination in cylindrical bodies.

## Graphical abstract

**Figure:**
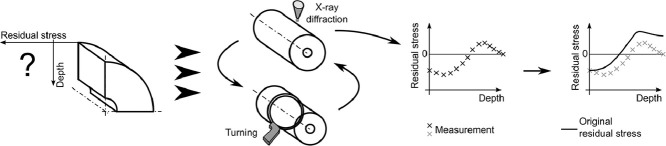

**Specifications table**

**Table:** 

Subject Area:	Engineering
More specific subject area:	*Residual stress determination in sizable cylindrical bodies*
Method name:	*Layer removal method for residual stress determination in the cylindrical bodies*
Name and reference of original method:	*M.G. Moore, W.P. Evans, Mathematical correction for stress in removed layers in X-ray diffraction residual stress analysis, SAE Tech. Pap. 45 (1958) 340–345*. 10.4271/580035. [2]
Resource availability:	*Any mathematical software can be used e.g. MATLAB, python …MATLAB function developed by the authors is available at:*https://ch.mathworks.com/matlabcentral/fileexchange/105690-layer-removal-residual-stress-determination-function*Necessary hardware: X-ray diffraction machine for the residual stress measurement; electrochemical etching machine; lathe*

## Background

**Fig. 1:**
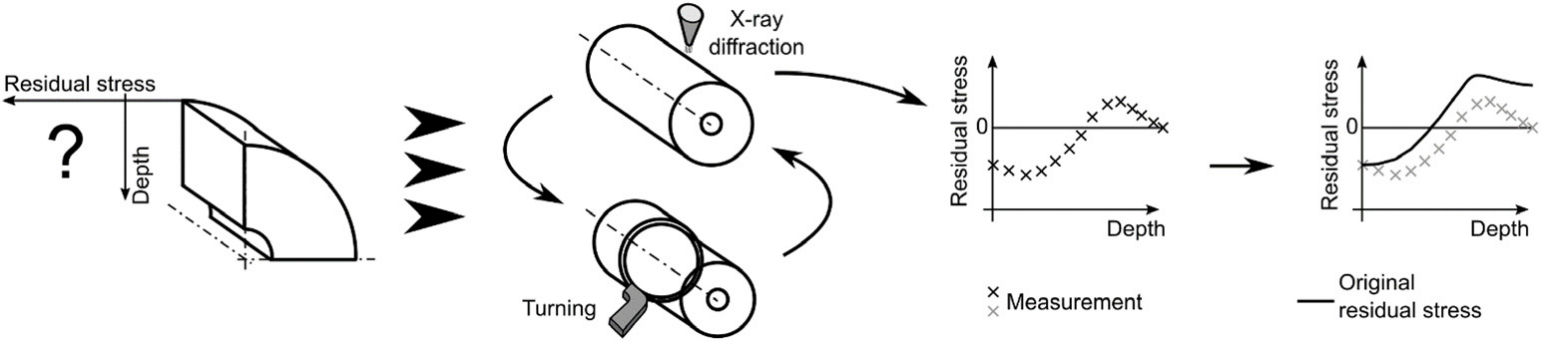
Schematic illustration of the residual stress determination process.

Residual stress is an elastic response [3,4] to non-uniform plastic deformation which is caused e.g. by surface finishing (like machining) or by material phase changes during a heat treatment. The X-ray diffraction method [[5], [6], [7], [8]] is widely used for residual stress measurement. However, it can measure only surface residual stress (a few micrometers under the investigated surface), which can be limiting in cases of sizable components, where it is necessary to measure the whole through-thickness distribution of residual stress. Therefore, repeated removal of the surface layer of the material and X-ray diffraction measurement is needed to determine the entire residual stress state in the sizable component, see Fig. 1. Since the residual stress is in equilibrium for the entire component, the removal causes residual stress redistribution. Therefore, after material removal, X-ray residual stress measurement results need to be corrected. Moore and Evans [2] in 1958 proposed a formula for such correction of values measured on cylindrical components that was based on the theory of elasticity. The presented paper proposes a new approach to the correction of X-ray diffraction data measured on cylindrical components, and it is applied to a specific case of investigating the residual stress state in railway axles. The new approach, referred to as the iterative evaluation method, is described here. It is considerably more accurate than Moore and Evans's solution in some cases.

## Method details

The iterative evaluation method is designed for the correction of residual stress measurement by X-ray diffraction on cylindrical components. The measurement itself must be carried out in the following steps:

1. Residual stress is measured by X-ray diffraction on the surface of a cylindrical body. Both tangential and axial residual stress are recorded.
2. Then, a surface material layer is removed from the cylindrical body by turning.
3. Residual stress is measured again on the newly created surface (again, both tangential and axial parts).
4. Steps 2 and 3 are repeated until only a small part of the original body remains.
  Note that thicknesses of the removed layers do not have to be uniform during the whole experiment. However, the typical thickness of the removed layer ranges from 1 to 5 mm for sizable components. It is generally better to plan a finer spacing in parts where larger gradients of residual stress can be expected.

Technical details of the X-ray diffraction technique are out of the scope of this paper. However, it should be mentioned that a certain preparation of the surface before the X-ray diffraction measurement itself is necessary. The machining itself produces residual stress, which should be removed by etching the machined surface. Typically, a layer of tens or hundreds of micrometers is removed to avoid the influence of machining on obtained values of residual stress.

The following conditions should be met when performing the X-ray diffraction measurement on a sizable cylindrical component to achieve the best results:

1. The measurement points should be at least one diameter away from the free end of the component – according to Saint-Venant's principle [9]. Hence, the body length should be ideally greater than two times the component diameter.
2. Residual stress distribution in the investigated part should be axisymmetric (the method described here is unsuitable for other cases).
3 Even the measured residual stress is zero, the residual stress measurement must be carried out in the whole cross-section and not only near the original outer surface.

After the experiment, there is a set of values of residual stress (axial and tangential) that were measured in certain positions in the cylinder (defined by radius *r*). However, these values are not the correct values of the original residual stress distribution. They were measured after disrupting the original state of equilibrium in the part by removing a layer of the material with the presence of residual stress (several times). These experimental results must be corrected by the numerical procedure. The correction procedure by the iterative evaluation method is described here.

The iterative procedure is based on repeatedly calculated values of an unbalance *U* and searching for such values of residual stress *σ* that will produce the value of *U* close to 0 for the whole component. Therefore, we are looking for the inner equilibrium state of the body. The measured values are used as starting points for the iterative process. The unbalance is calculated as

(1) $$U=\frac{\sum_{i=1}^{n}F_{dir,i}}{S_{dir,\;tot}}=\frac{\sum_{i=1}^{n}S_{dir,i}\sigma_{dir,i}}{S_{dir,\;tot}},$$

where *F* is reaction force, *S* corresponding area, *σ* is stress, *n* is a number of measured depth points + 1, the *dir* subscript corresponds to the evaluated direction (*dir = ax* for axial and *dir = tg* for tangential), and the *i* subscript corresponds to the radius of the evaluated surface (*i* = *r_in_* corresponds to the inner surface, *r_ou_* is the outer surface radius and *r*_1_ to *r_n_* are the radii in between).

Two assumptions are made at the beginning of the determination process:

1. A theoretical value of 0 MPa is added to the measured values for the inner radius, assuming that after removing all material (which is physically impossible), 0 MPa, would be measured. This value is added to estimate residual stress in the region, followed by the final physically possible measurement. If there is no hole in the cylinder (solid cylinder), 0 MPa value corresponds to radius *r_in_* = 0 mm.
2. The linear residual stress distribution is assumed between each measured point.

**Fig. 2:**
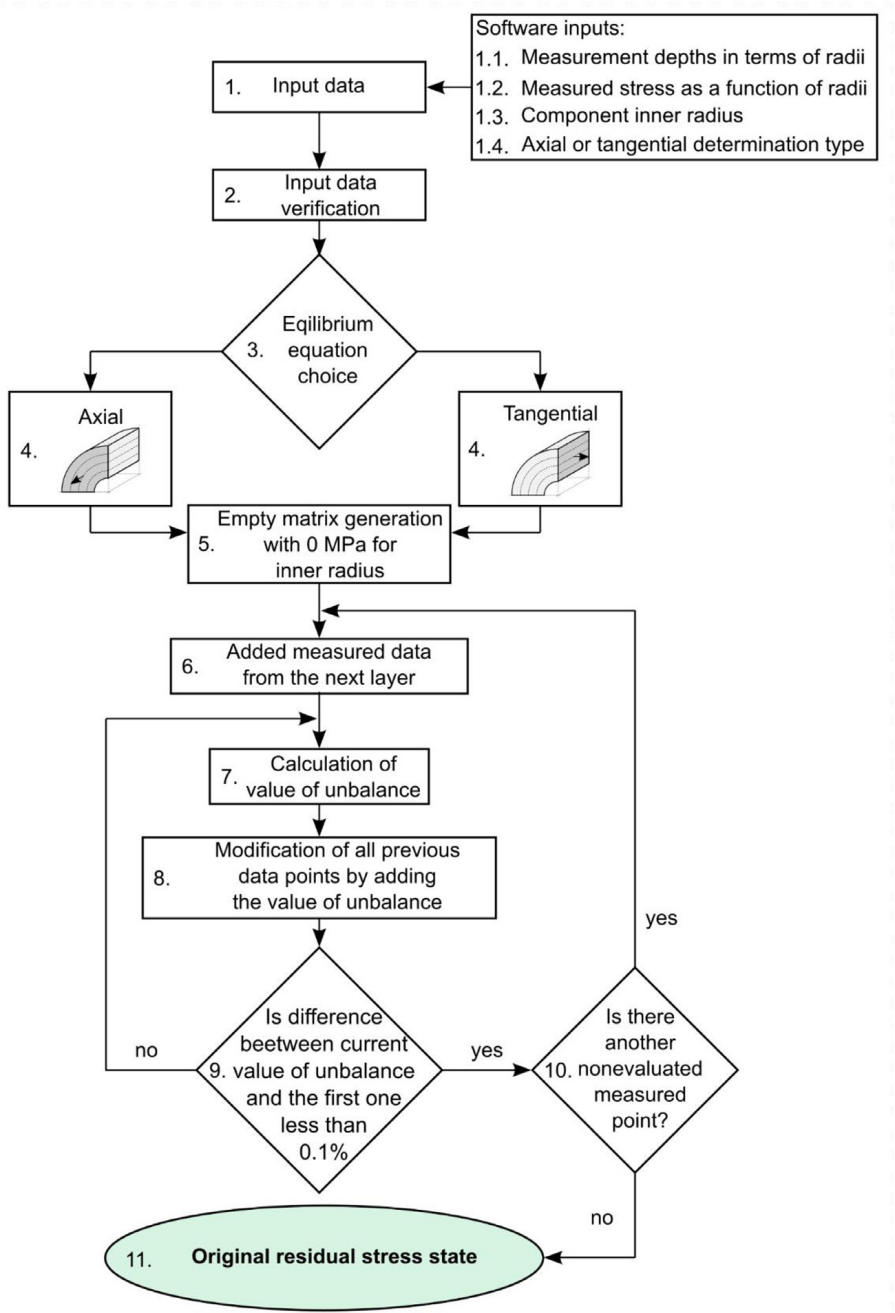
Scheme of the iterative evaluation method for the correction of residual stress values measured by X-ray diffraction.

The whole procedure was programmed as a MATLAB function. Fig. 2 shows the scheme of the procedure.

The determination process itself runs in two nested cycles. The outer cycle determines the portion of layers for the inner cycle to work on, starting from the innermost layer and adding one layer in each step. The inner cycle evaluates the force equilibrium condition for the current portion of layers. It is important to note that the layers must correspond precisely to the removed layers when performing the measurements.

**Fig. 3:**
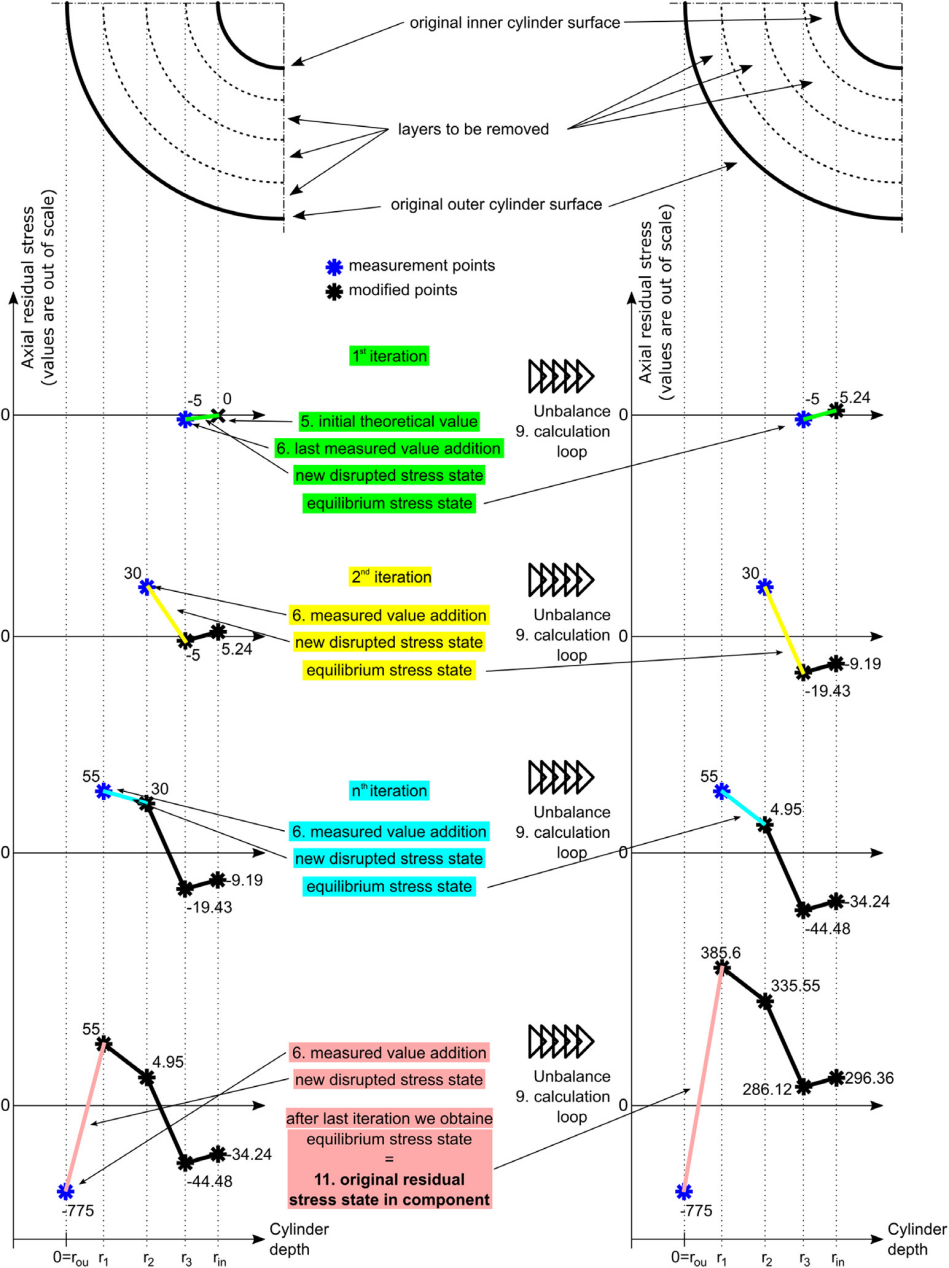
Schematic illustration of the analytical residual stress determination process.

Fig. 3 illustrates the evaluation process. The blue points in the figure stand for the measured values. The black X point is the additional theoretical value of 0 MPa. The iterations run as follows:

**1st iteration**

**Fig. 4:**
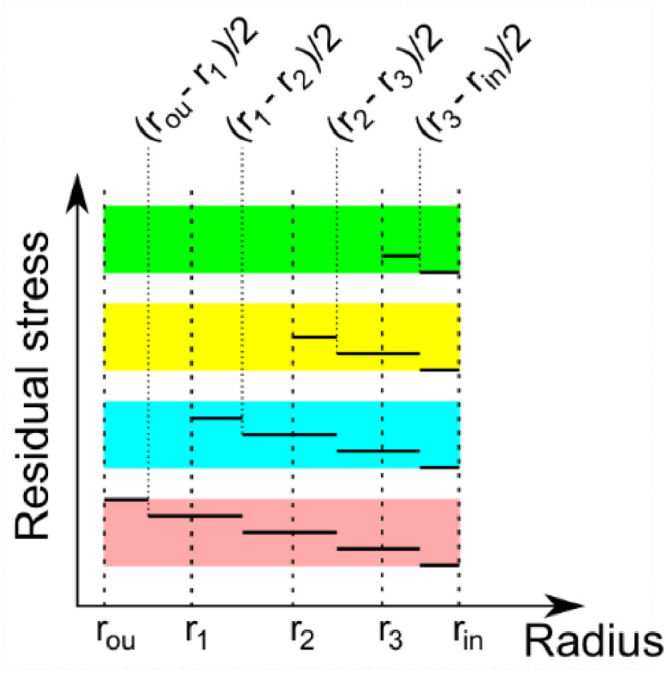
Corresponding area functions behavior.

The determination starts from the innermost layer (the interval from *r_in_* to *r*_3_), which is bounded by the 0 MPa at *r_in_* and the last measured point at *r*_3_. Then, the inner circle works on this interval only. It calculates the value of unbalance *U*, and then shifts the values of stress by the calculated unbalance and calculates the unbalance again until it reaches the point when the *U* < 0.1% (chosen calculation error) of the first unbalance (this condition is referred to as the *equilibrium condition* in further text). However, the measured value at *r*_3_ cannot be changed at this point. It is regarded as the boundary condition. The only value that can be shifted is the value of 0 MPa. The equilibrium equation for the first iteration in the case of axial residual stress evaluation is:

(2) $$U=\frac{\sum_{i=1}^{n=2}F_{dir,i}}{S_{dir,\;tot}}=\frac{\pi \left(r_{3}^{2}-{\left(r_{in}+\frac{r_{3}-r_{in}}{2}\right)}^{2}\right)\sigma_{ax,\;r_{3}}+\pi \left({\left(r_{in}+\frac{r_{3}-r_{in}}{2}\right)}^{2}-r_{in}^{2}\right)\sigma_{ax,\;r_{in}}}{\pi \left(r_{3}^{2}-r_{in}^{2}\right)}$$

where *r*_3_ is the radius of the last measured point and *r_in_* is zero for the solid cylinder and inner radius for the hollow cylinder. To consider the linear stress distribution between the measured points, the area corresponding to the evaluated point must be equal to half of the current layer thickness. Fig. 4 shows corresponding areas for the schematic case shown in Fig. 3.

The next iteration starts when the equilibrium condition is fulfilled.

**2nd iteration**

The next layer is added to the innermost layer to form the next interval for the equilibrium search. In the 2nd iteration, the interval is from *r_in_* to *r*_2_. Now, the measured value at *r*_2_ is the boundary condition that cannot be changed. The previous values at *r*_3_ and *r_in_* must be changed now to find the equilibrium state. This is done again by shifting the values by the newly calculated value of unbalance until the equilibrium condition is met (*U* < 0.1% of the first calculated unbalance).

**nth iteration**

In every following iteration, the process of adding the next layer and searching for the equilibrium by shifting the previously calculated values is repeated until every measured depth is used and the final equilibrium state is obtained.

The procedure of the iterative evaluation method is illustrated in this article using a fictive set of measured values. The values were chosen arbitrarily. The shape of the distribution is based on typical residual stress distribution in hardened railway axles. A railway axle is a typical example of a bulky cylindrical component, where it is required to describe the residual stress accurately.

**Fig. 5:**
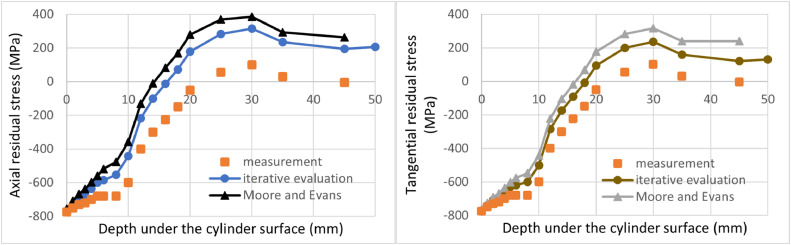
Comparison between the proposed iterative evaluation method and Moore and Evans solution while considering all measured positions. Axial residual stress on the left, tangential residual stress on the right.

The showcase considers a cylindrical component with an outer diameter of 200 mm and an inner diameter of 100 mm with sufficient length according to the first experimental assumption. The measurements were considered with steps (thickness of machined layers) of 1 mm from 0 to 6 mm depth, with steps of 2 mm from 6 to 20 mm depth, and with steps of 5 mm from 20 to 45 mm depth. Together it represents 18 removed layers, i.e., measured points. The imaginary residual stress measurements are summarized in Table 1 (in the Supplementary material), and Fig. 5 shows results from imaginary residual stress measurement. The original equilibrium stress states calculated from the measured values by the Moore and Evans' method and by the iterative evaluation method are plotted by the solid lines. There is a large discrepancy between the measured values and the equilibrium state (determined by either method), which illustrates the importance of performing of described a numerical correction.

**Fig. 6:**
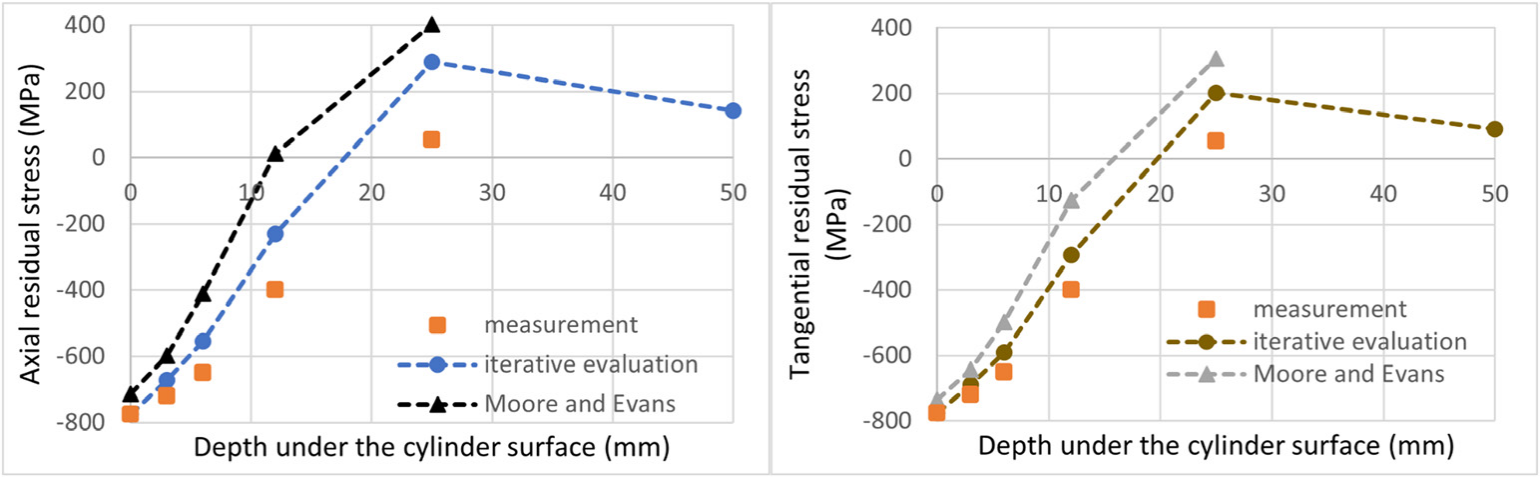
Comparison between the proposed iterative evaluation method and Moore and Evans solution while considering a coarse sampling. Axial residual stress on the left, tangential residual stress on the right.

Fig. 5 and Fig. 6 also compare the presented iterative evaluation method and previous Moore and Evans' solutions. Fig. 5 represents the case where a fine depth sampling was designed and the experimental procedure correctly performed. Axial residual stress results near the outer surface are close to each other. The surface value for the iterative evaluation method is equal to the measured value -775 MPa at the cylindrical surface without any machining, whereas Moore and Evans's solution evaluates surface value as -754 MPa. The difference is 2.6%. The difference in this point can be neglected because it is still small compared to the usual overall measurement accuracy of the X-ray diffraction. However, in the case of the maximal axial residual stress, the iterative evaluation method results show the value of 316 MPa, while Moore and Evans' method produces 386 MPa. The difference is there 22%, which is not negligible. Fig. 6 represents the case of the experiment, where coarse layer spacing was chosen, and measurement was interrupted after the first tensile residual stress value was obtained. In this case, the difference in terms of the axial residual stress for the surface values is 8%, and for the maximum obtained tensile residual stress represents 39%.

**Fig. 7:**
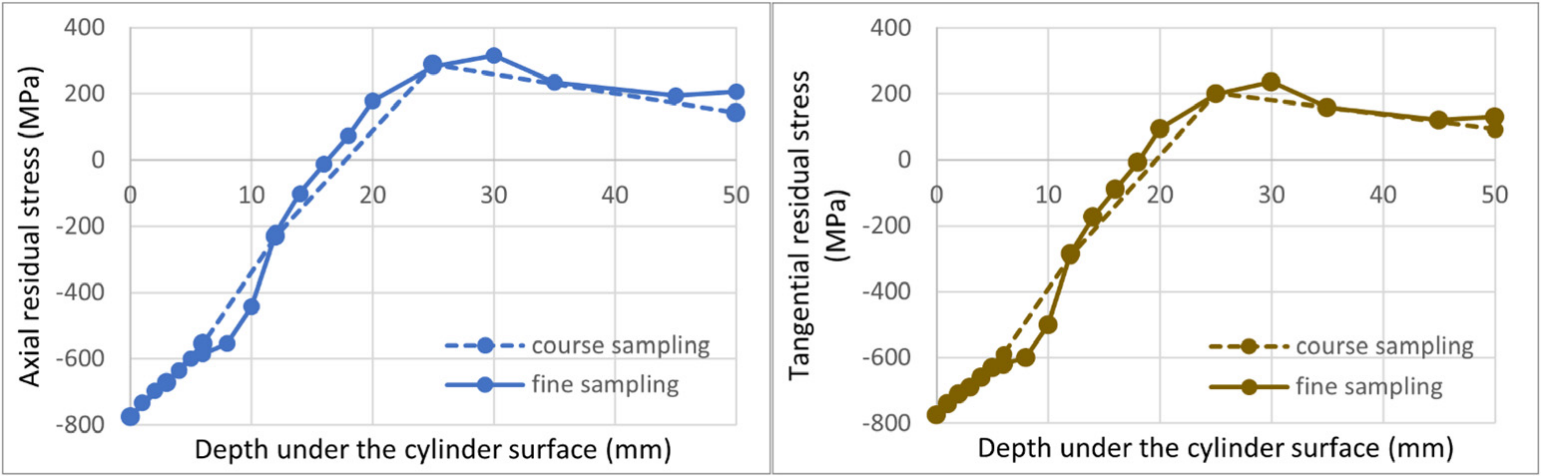
Comparison of the proposed iterative evaluation method while considering all measured points (fine sampling) and coarse sampling. Axial residual stress on the left, tangential residual stress on the right.

**Fig. 8:**
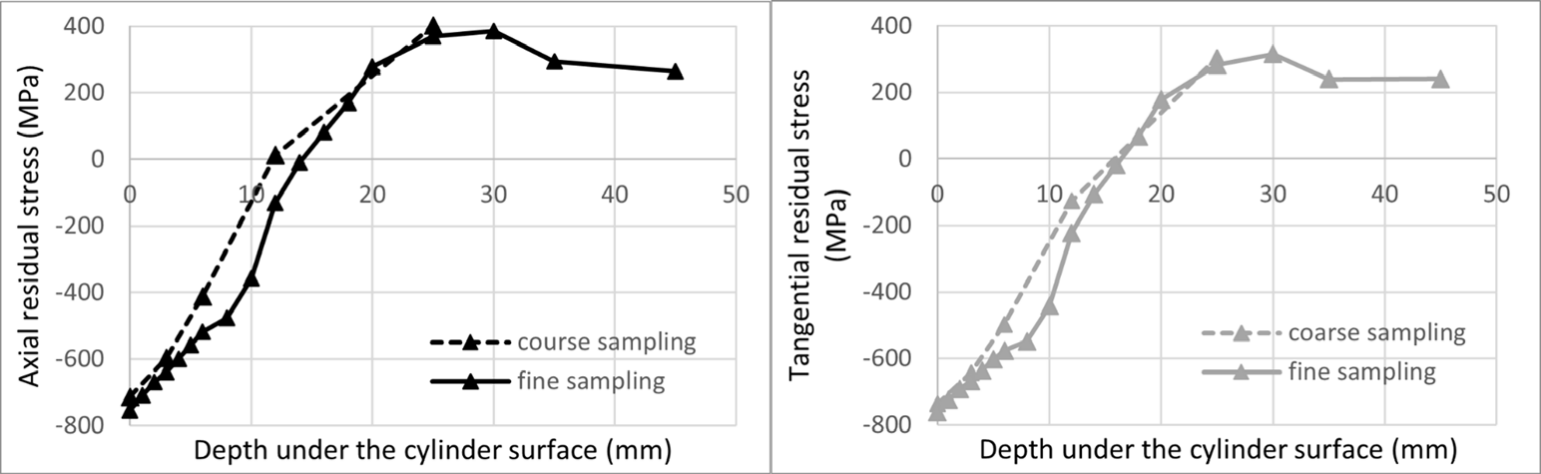
Comparison of Moore and Evans's solution while considering all measured points (fine sampling) and a coarse sampling. Axial residual stress on the left, tangential residual stress on the right.

Fig. 7 a Fig. 8 compare solutions obtained by the proposed iterative evaluation method and Moore and Evans' method, respectively, for fine and coarse sampling. One can see that less frequent measurement points are not an issue for the iterative evaluation method. Evaluated points lie on top of each other. For Moore and Evans solution, the values show a very good match. However, they differ in some cases.

**Fig. 9:**
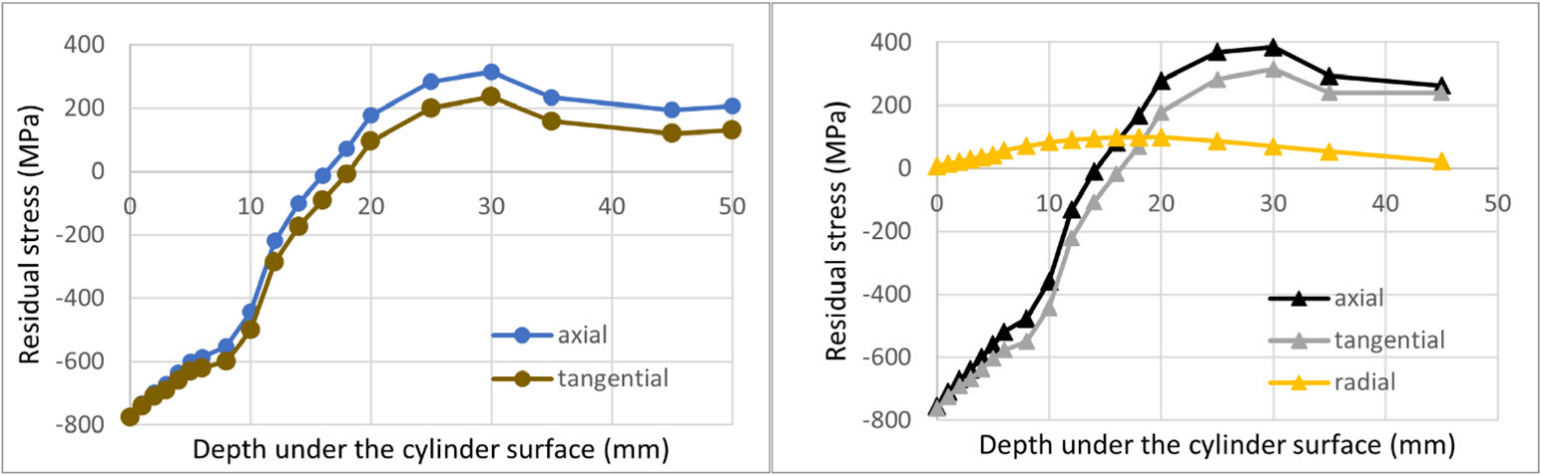
Finite residual stress results obtained by the proposed iterative evaluation method on the left and by the Moore and Evans solution on the right.

Fig. 9 shows all the evaluated data for the iterative evaluation method's and Moore and Evans' solutions. The advantage of the iterative evaluation method is the possibility to evaluate the whole cross-section of the cylinder. On the other hand, Moore and Evans's solution can obtain radial residual stress.

Table 1 (in the Supplementary material) contains all the data presented in this paper. Measurement points can be used for the validation in case of own procedure development.

## Declaration of Interests

The authors declare that they have no known competing financial interests or personal relationships that could have appeared to influence the work reported in this paper.

## Appendix A Supplementary materials

application
