# Supplementary material for: Residual stress determination by the layer removal and X-ray diffraction measurement – correction method
Source: MethodsX. 2022 Jun 22;9:101768. doi: 10.1016/j.mex.2022.101768 (PMC9253484; doi:10.1016/j.mex.2022.101768)
Supplement: Supplementary file 1 [file mmc1.docx]

**Supplementary material:**

Table 1 Data used for the evaluation and results obtained by both methods

| depth (mm) | r (mm) | axial and tangential measurement (MPa) | axial residual stress (MPa) | | tangential residual stress (MPa) | | radial residual stress (MPa) | |
| --- | --- | --- | --- | --- | --- | --- | --- | --- |
|  |  |  | iterative evaluation method | Moore and Evans | iterative evaluation method | Moore and Evans | iterative evaluation method | Moore and Evans |
| 0 | 100 | -775 | -775.0 | -754.3 | -775.0 | -762.1 | - | 7.8 |
| 1 | 99 | -750 | -733.6 | -709.0 | -739.8 | -724.3 | - | 15.3 |
| 2 | 98 | -730 | -697.6 | -668.9 | -709.7 | -691.5 | - | 22.6 |
| 3 | 97 | -720 | -671.7 | -638.6 | -689.7 | -668.5 | - | 29.9 |
| 4 | 96 | -700 | -635.9 | -598.6 | -659.8 | -635.6 | - | 36.9 |
| 5 | 95 | -680 | -600.4 | -558.8 | -630.0 | -602.6 | - | 43.8 |
| 6 | 94 | -680 | -584.9 | -518.5 | -620.2 | -576.4 | - | 57.9 |
| 8 | 92 | -680 | -553.4 | -476.5 | -600.2 | -548.2 | - | 71.7 |
| 10 | 90 | -600 | -442.9 | -357.9 | -500.8 | -441.6 | - | 83.7 |
| 12 | 88 | -400 | -218.5 | -131.1 | -285.2 | -222.1 | - | 91.0 |
| 14 | 86 | -300 | -101.0 | -10.0 | -173.9 | -106.0 | - | 96.0 |
| 16 | 84 | -225 | -12.4 | 81.6 | -90.1 | -17.4 |  | 99.0 |
| 18 | 82 | -150 | 72.5 | 168.2 | -8.7 | 68.3 | - | 100.0 |
| 20 | 80 | -50 | 178.0 | 278.5 | 94.9 | 178.4 | - | 100.1 |
| 25 | 75 | 55 | 282.8 | 370.3 | 199.7 | 282.7 | - | 87.6 |
| 30 | 70 | 100 | 315.7 | 386.1 | 236.5 | 316.0 | - | 70.1 |
| 35 | 65 | 30 | 234.4 | 293.5 | 158.9 | 239.7 | - | 53.8 |
| 45 | 55 | -5 | 194.1 | 263.7 | 120.3 | 240.4 | - | 23.3 |
| 50 | 50 | - | 206.4 | - | 130.3 | - | - | - |

**MATLAB LRRSD.m file:**

function [result] = LRRSD(varargin)

% LRRSD Layer Removal Residual Stress Determination

% Residual stress determination by the Layers removal-based method.

%

% S_real = LRRSD(R,S,Rm,type)

% R - is a vector. Use here values of the measurement points radii

% sorted in descending order. The last radius point is for the last

% point truly measured.

% S - is a vector. Use here values of the measured residual stress

% for previously specified radii.

% Rm - is scalar. It is the inner radius of the cylinder component.

% If cylinder component is without a hole, use Rm = 0.

% type - is a string. Use "ax" for axial/longitudinal residual stress or

% use "tg" for tangential/transversal residual stress determination.

%

%===========================================================================================

% Authors: Pavol Dlhý, Jan Poduška, Pavel Pokorný, Michal Jambor, Luboš Náhlík, Pavel Hutař

% Affiliation: Institute of Physics of Materials of the Czech Academy of Sciences, v.v.i.

% E-mail: dlhy@ipm.cz; nahlik@ipm.cz

% Version: 1.0

% Last modified: 13.01.2022

% Developed in MATLAB R2019b

%===========================================================================================

narginchk(4,4)

[R_cylinder,Stress,R_min,type] = parseinputs(varargin{:});

if ~isnumeric(R_cylinder) || ~isnumeric(Stress) || ~isnumeric(Stress)

error('Input must be numeric.')

end

[a, b] = size(R_cylinder);

if ~(a == 1 || b == 1)

error('The first input must be a vector.')

end

if b > a

R_cylinder = R_cylinder';

end

if R_cylinder(1,1) < R_cylinder(end,1)

error('The first input must be the radii of the measured cylinders in descending order.')

end

if ~all(R_cylinder > 0)

error('Radii of the measured points must be greater than zero.')

end

[c, d] = size(Stress);

if ~(c == 1 || d == 1)

error('Second input must be vector.')

end

if d > c

Stress = Stress';

end

if R_min == R_cylinder(end)

error('Do not use the Rm value for measured radii.')

end

if length(R_cylinder)~=length(Stress)

error('R and S vectors are not the same length.')

end

[e, f] = size(R_min);

if ~(e == 1 && f == 1)

error('The third input must be scalar.')

end

g = {'ax','tg'};

if ~sum(strcmp(type,g))

error('Wrong type choice. Use only "ax" or "tg" for axial or tangential residual stress determination, respectively.')

end

%% Axial stress

if strcmp(type,'ax')

RS = [R_cylinder, Stress];

RS = [RS; R_min 0];

rows = length(RS(:,1));

S_ax_original = zeros(rows,rows-1);

mm = 1;

for ii = 1:rows-1

S_ax_original(end-ii,mm) = RS(end-mm,2);

mm = mm + 1;

end

R1 = RS(end,1);

R2 = RS(end-1,1);

S2_ax = RS(end-1,2);

S_ax_original(rows,1) = -(S2_ax*(R2^2-(R2-(R2-R1)/2)^2))/((R1+(R2-R1)/2)^2-R1^2);

mm = 2;

for ii = 2:rows-1

S_ax_help = S_ax_original(:,ii-1);

S_ax_help = S_ax_help + S_ax_original(:,ii);

lgc = logical(S_ax_help);

for nn = 1:10

posun = SUBF_ax_equil(RS(lgc,1),S_ax_help(lgc));

if abs(posun) < 0.001

break

end

S_ax_help(lgc) = S_ax_help(lgc) - posun;

for bb = 1:length(lgc)

if lgc(bb) == 1

S_ax_help(bb) = RS(end-mm,2);

break

end

end

end

S_ax_original(:,ii) = S_ax_help;

mm = mm + 1;

end

% Result

result = S_ax_original(:,end);

end

%% Tangential stress

if strcmp(type,'tg')

RS = [R_cylinder, Stress];

RS = [RS; R_min 0];

rows = length(RS(:,1));

S_tg_original = zeros(rows,rows-1);

mm = 1;

for ii = 1:rows-1

S_tg_original(end-ii,mm) = RS(end-mm,2);

mm = mm + 1;

end

R1 = RS(end,1);

R2 = RS(end-1,1);

S2_tg = RS(end-1,2);

S_tg_original(rows,1) = -(S2_tg*(R2-(R2-(R2-R1)/2)))/((R1+(R2-R1)/2)-R1);

mm = 2;

for ii = 2:rows-1

S_tg_help = S_tg_original(:,ii-1);

S_tg_help = S_tg_help + S_tg_original(:,ii);

lgc = logical(S_tg_help);

for nn = 1:10

posun = SUBF_tg_equil(RS(lgc,1),S_tg_help(lgc));

if abs(posun) < 0.001

break

end

S_tg_help(lgc) = S_tg_help(lgc) - posun;

for bb = 1:length(lgc)

if lgc(bb) == 1

S_tg_help(bb) = RS(end-mm,2);

break

end

end

end

S_tg_original(:,ii) = S_tg_help;

mm = mm + 1;

end

% Result

result = S_tg_original(:,end);

end

end

%% subfunctions

function [subf_gap] = SUBF_ax_equil(subf_R_cylinder,subf_Stress)

subf_RS = [subf_R_cylinder, subf_Stress];

[subf_m,]=size(subf_RS);

subf_suma_Nap = 0;

subf_R_vonk = subf_RS(1,1);

subf_R_vnut = subf_RS(2,1);

subf_S_axi = subf_RS(1,2);

subf_suma_Nap = subf_suma_Nap + pi*(subf_R_vonk^2-(subf_R_vonk-(subf_R_vonk-subf_R_vnut)/2)^2)*subf_S_axi;

subf_R_vonk = subf_RS(end-1,1);

subf_R_vnut = subf_RS(end,1);

subf_S_axi = subf_RS(end,2);

subf_suma_Nap = subf_suma_Nap + pi*((subf_R_vnut+(subf_R_vonk-subf_R_vnut)/2)^2-subf_R_vnut^2)*subf_S_axi;

for i=2:subf_m-1

subf_R_vonk = subf_RS(i-1,1);

subf_R_stre = subf_RS(i,1);

subf_R_vnut = subf_RS(i+1,1);

subf_S_axi = subf_RS(i,2);

subf_suma_Nap = subf_suma_Nap + pi*((subf_R_stre+(subf_R_vonk-subf_R_stre)/2)^2-(subf_R_stre-(subf_R_stre-subf_R_vnut)/2)^2)*subf_S_axi;

end

subf_suma_Plo = pi*(subf_RS(1,1)^2-subf_RS(end,1)^2);

subf_gap=subf_suma_Nap/subf_suma_Plo;

end

function [subf_gap] = SUBF_tg_equil(subf_R_cylinder,subf_Stress)

subf_RS = [subf_R_cylinder, subf_Stress];

[subf_m,]=size(subf_RS);

subf_suma_Nap = 0;

subf_R_vonk = subf_RS(1,1);

subf_R_vnut = subf_RS(2,1);

subf_S_tan = subf_RS(1,2);

subf_suma_Nap = subf_suma_Nap + (subf_R_vonk-(subf_R_vonk-(subf_R_vonk-subf_R_vnut)/2))*subf_S_tan;

subf_R_vonk = subf_RS(end-1,1);

subf_R_vnut = subf_RS(end,1);

subf_S_tan = subf_RS(end,2);

subf_suma_Nap = subf_suma_Nap + ((subf_R_vnut+(subf_R_vonk-subf_R_vnut)/2)-subf_R_vnut)*subf_S_tan;

for i=2:subf_m-1

subf_R_vonk = subf_RS(i-1,1);

subf_R_stre = subf_RS(i,1);

subf_R_vnut = subf_RS(i+1,1);

subf_S_tan = subf_RS(i,2);

subf_suma_Nap = subf_suma_Nap + ((subf_R_stre+(subf_R_vonk-subf_R_stre)/2)-(subf_R_stre-(subf_R_stre-subf_R_vnut)/2))*subf_S_tan;

end

subf_suma_Plo = (subf_RS(1,1)-subf_RS(end,1));

subf_gap=subf_suma_Nap/subf_suma_Plo;

end

function [subf_R_cylinder,subf_Stress,subf_R_min,subf_type] = parseinputs(varargin)

subf_R_cylinder = varargin{1};

subf_Stress = varargin{2};

subf_R_min = varargin{3};

subf_type = varargin{4};

end
